# Pathways through opiate use and offending: A systematic review

**DOI:** 10.1016/j.drugpo.2016.08.015

**Published:** 2017-01

**Authors:** Karen P. Hayhurst, Matthias Pierce, Matthew Hickman, Toby Seddon, Graham Dunn, John Keane, Tim Millar

**Affiliations:** a Centre for Mental Health and Safety, Institute of Brain, Behaviour and Mental Health, The University of Manchester, Manchester M13 9PL, UK; b NIHR Health Protection Research Unit in Evaluation, School of Social and Community Medicine, The University of Bristol, Bristol BS8 2PS, UK; c School of Law, The University of Manchester, Manchester M13 9PL, UK; d Centre for Biostatistics, Institute of Population Health, The University of Manchester, Manchester M13 9PL, UK; e School of Computer Science and Manchester Institute of Biotechnology, The University of Manchester, Manchester M13 9PL, UK

**Keywords:** Crime, Opiate use, Substance abuse, Systematic review

## Abstract

**Background:**

Although evidence points to a strong link between illicit drug use and crime, robust evidence for temporal order in the relationship is scant. We carried out a systematic review to assess the evidence for pathways through opiate/crack cocaine use and offending to determine temporal order.

**Methods:**

A systematic review sourced five databases, three online sources, bibliographies and citation mapping. Inclusion criteria were: focus on opiate/crack use, and offending; pre-drug use information; longitudinal design; corroborative official crime records. Rate ratios (RR) of post-drug use initiation to pre-drug use initiation were pooled using random effects meta-analysis.

**Results:**

20 studies were included; UK (9) and US (11). All were of opiate use. Mean age at (recorded) offending onset (16.7 yrs) preceded mean age at opiate-use onset (19.6 yrs). Substantial heterogeneity (over 80%: unexplained by meta-regression) meant that RRs were not pooled. The RR for total (recorded) offending ranged from 0.71 to 25.7 (10 studies; 22 subsamples: positive association, 4: equivocal, 1: negative association). Positive associations were observed in 14/15 independent samples; unlikely to be a chance finding (sign test *p* = 0.001).

Individual offence types were examined: theft (RR 0.63–8.3, 13 subsamples: positive, 9: equivocal, 1 negative); burglary (RR 0.74–50.0, 9 subsamples: positive, 13: equivocal); violence (RR 0.39–16.0, 6 subsamples: positive, 15: equivocal); and robbery (RR 0.50–5.0, 5 subsamples: positive, 15: equivocal).

**Conclusions:**

Available evidence suggests that onset-opiate use accelerates already-existing offending, particularly for theft. However, evidence is out of date, with studies characterised by heterogeneity and failure to use a matched non-opiate-user comparison group to better-establish whether onset-opiate use is associated with additional crime.

## Introduction

Drugs policy over the last two decades in the UK, and other countries, such as the US and Australia, has been very strongly influenced by the assumed link between drugs and crime (HM Government, 2010, Home Office, 2011, Home Office, 2016) and the idea that tackling drug use will affect crime. Existing evidence suggests a strong link between illicit drug use and involvement in crime (Bennett, Holloway, & Farrington, 2008), especially income-generating, acquisitive crime (Bukten et al., 2011b). The association has been observed primarily among arrestees (Pierce et al., 2015b), prisoners (Johnson, 2006) and people entering drug treatment (Darke et al., 2009). However, these groups may not be representative of the wider drug-using population as offending rates can be atypical in the period immediately prior to arrest, imprisonment or treatment (McGlothlin, Anglin, & Wilson, 1978); and drug-using offenders may be more likely than non-using offenders to be apprehended (Bond & Sheridan, 2007).

The drug-crime link appears particularly compelling among individuals with frequent and problematic use of opiates, such as heroin (Kaye, Darke, & Finlay-Jones, 1998), and/or crack cocaine (Bennett et al., 2008, Comiskey et al., 2012). Evidence synthesis concludes that the odds of offending are six times greater for crack users than non-crack users and three times greater for heroin users than non-heroin users (Bennett et al., 2008). Opiate/crack users comprise 81% of those in receipt of structured drug treatment services in England and are the group predominantly targeted by policy initiatives to divert drug-using offenders into treatment (Home Office, 2011, PHE, 2014). For these reasons, our review focuses on opiate/crack users.

The drug-crime association is supported by studies indicating the impact of periods of addiction versus non-addiction on offending (Nurco, Shaffer, Ball, & Kinlock, 1984). Receipt of drug treatment is associated with reduced offending (Bukten et al., 2011a) and higher offending rates are associated with more serious drug use (Hammersley, Forsyth, Morrison, & Davies, 1989).

Studies examining gender differences in the opiate use–crime relationship, in particular, tend to agree that males: are younger at crime onset (Farabee, Joshi, & Anglin, 2001), commit a higher volume of crime (Hall, Bell, & Carless, 1993) and initiate opiate use at a younger age than females (Farabee et al., 2001). However, while females appear more likely to proceed from opiate-use onset to crime (Swan & Goodman-Delahunty, 2013), crime tends to precede opiate use for males (Kaye et al., 1998). Some studies point to a higher likelihood of offending in female drug users than in male drug users (Bennett et al., 2008, Pierce et al., 2015b).

There is continued debate about whether the existing evidence is sufficiently robust to indicate a causal relationship between drug use and crime (Seddon, 2000), although much of UK drug policy is explicitly grounded in the existence of a causal link (HM Government, 2010, Home Office, 2011, Home Office, 2016). One theory for the associative pathway between opiate use and crime is the need to generate income to fund expensive drug use (Bennett et al., 2008, Goldstein, 1985). However, Hayhurst et al. (2013) have demonstrated elsewhere that behavioural and demographic factors are associated more strongly with acquisitive crime than drug use expenditure. Further, although a small proportion of opiate-using offenders commit a high volume of crime (Bukten et al., 2011b) some commit no crime (Nurco, Hanlon, Balter, Kinlock, & Slaght, 1991). Other theories imply that the pathway reflects that illegality of drug use promotes opportunities for further involvement with criminal networks (Hammersley et al., 1989). A further theory proposes that the pathway results from the psychopharmacological effects of drug use on behaviour. For example, ingestion of stimulant psychoactive substances, such as crack cocaine, has been linked to violent criminal behaviour (Brownstein, 2016). Furthermore, the observed association itself may be spurious (Altman, 1991), i.e. due to a third extraneous factor, or common cause, separately implicated in both opiate/crack use and offending, for example, socioeconomic factors (Gauffin, Vinnerljung, Fridell, Hesse, & Hjern, 2013) or personality disorder (Shand, Slade, Degenhardt, Baillie, & Nelson, 2011).

The establishment of a causal relationship between drug use and crime requires evidence of temporal order in addition to evidence of an association. The observed association between the putative independent variable (drug use) and the dependent variable (crime) does not necessarily imply causation (Altman, 1991). The establishment of causation requires evidence that the cause (drug use) precedes the effect (crime) in the pathway. The existing evidence appears limited in its capacity to establish temporal order in the drug use–crime relationship; i.e. is opiate/crack-use onset more likely to precede offending or vice versa? Existing evidence is inconsistent in its choice of crime categories: some studies do not differentiate crime by type (e.g. Stenbacka & Stattin, 2007). Others group dissimilar drugs when examining the impact of drug use on crime and/or include alcohol in measures of substance misuse (e.g. Larm, Hodgins, Larsson, Samuelson, & Tengstrom, 2008). Existing literature is limited in its coverage of the pre-addiction period, despite recommendations from almost 40 years ago that research should focus on “criminal behaviour prior to the onset of use” (Research Triangle Institute, 1976).

Evidence synthesis of studies focusing specifically on pathways through drug use and offending is absent. Much of the review literature concentrates on the effectiveness of criminal justice responses to existing offending by drug users (Hayhurst et al., 2015, Perry et al., 2009, Perry et al., 2015) or focuses on the strength of the relationship between existing drug use and crime (Bennett et al., 2008).

We set out to clarify the strength of the evidence in this area and whether, and to what extent, available evidence reflects current patterns of behaviour and supports current policy responses. We conducted a systematic review with three main aims: (1) to explore the temporal order between opiate/crack use and crime initiation in studies examining the pathway through opiate/crack use and offending; (2) to examine the impact of opiate/crack-use onset on crime rates; and (3) to examine factors that might influence the relationship between opiate/crack-use onset and crime, for example, gender.

## Methods

Systematic review methods were based on the PRISMA (Preferred Reporting Items for Systematic Reviews and Meta-Analyses) statement (Moher, Liberati, Tetzlaff, Altman, & Group, 2009).

### Study identification

Relevant studies were identified via electronic databases, online sources and bibliography screening. Comprehensive search terms, comprising medical subject headings, thesaurus terms and text words derived from titles and keywords of published literature, were developed using the Applied Social Sciences Index and Abstracts (ASSIA) database. The full search strategy is available online (Supplementary Table 1). The search string was applied to the following databases: ASSIA; Social Services Abstracts; Sociological Abstracts; International Bibliography of the Social Sciences (IBSS); and National Criminal Justice Reference Service (NCJRS) with databases searched since inception. Supplementary searches were performed on PubMed, DrugScope and UK Home Office online resources. Bibliographies of retrieved manuscripts were screened and citation mapping (ISI Web of Science) identified further publications cited by included studies. Searches were completed in June 2014. No setting, date or geographical restrictions were applied; searches were limited to English language sources.

### Inclusion and eligibility

Included studies met the following criteria: focus on use of opiates and/or crack cocaine (the population of interest); focus on criminal offending (the behaviour of interest); pre-opiate/crack-use initiation information (necessary to establish temporal order); findings from longitudinal studies with corroborative official crime records (necessary to establish developmental causality in offending and avoid recall bias); and findings from primary data collection or systematic review. Non-peer-reviewed findings reported solely in books, conference proceedings, dissertations or theses were excluded. The inclusion criteria were applied to titles and abstracts of retrieved studies; the full text of those potentially relevant was screened independently by two reviewers, with 100 per cent agreement following discussion, although a third reviewer was available to resolve disagreements. Risk of bias was assessed by the Newcastle-Ottawa quality assessment scale for cohort studies (Wells & Shay, 2000). This scale is recommended for quality assessment in epidemiological systematic reviews (Deeks et al., 2003) and is regularly used in systematic reviews of observational health studies (e.g. Asbridge, Hayden, & Cartwright, 2012). Content/face validity and inter-rater reliability have been established. The Newcastle–Ottawa scale comprises eight items covering appropriateness of selection criteria used (maximum score 4), comparability of participant groups (maximum score 2) and assessment of outcome (maximum score 3); a higher score (out of a possible 9) indicates lower risk of bias (Deeks et al., 2003).

### Data synthesis

Data were extracted from included studies via a piloted data extraction form and verified by a second reviewer. The following data were extracted: study characteristics (Table 1, Table 2); average age at opiate/crack onset; average age at (recorded) offending onset (Table 3); and offending rates (Table 4, Table 5). Offending rates were grouped according to offence type. Rate ratios (RR) of post-use initiation to pre-use initiation were calculated. The log of the RR was pooled via meta-analysis using random effects models. Meta-analysis is the statistical synthesis of results from a series of studies; random effects are chosen where variation among studies is expected (Borenstein, Hedges, Higgins, & Rothstein, 2009). Heterogeneity was assessed using Chi^2^ and I^2^ statistics. Funnel plots were interpreted visually to assess the extent of possible publication bias. Meta-analysis was carried out via Review Manager (version 5.3).

## Results

Searches resulted in 5204 hits: ASSIA (1530); Social Services Abstracts (879); Sociological Abstracts (1601); IBSS (389); and NCJRS (321). Supplementary searches resulted in additional hits: PubMed (114); DrugScope (81); UK Home Office (5); bibliography search (190); and citation mapping (94).

**Fig. 1:**
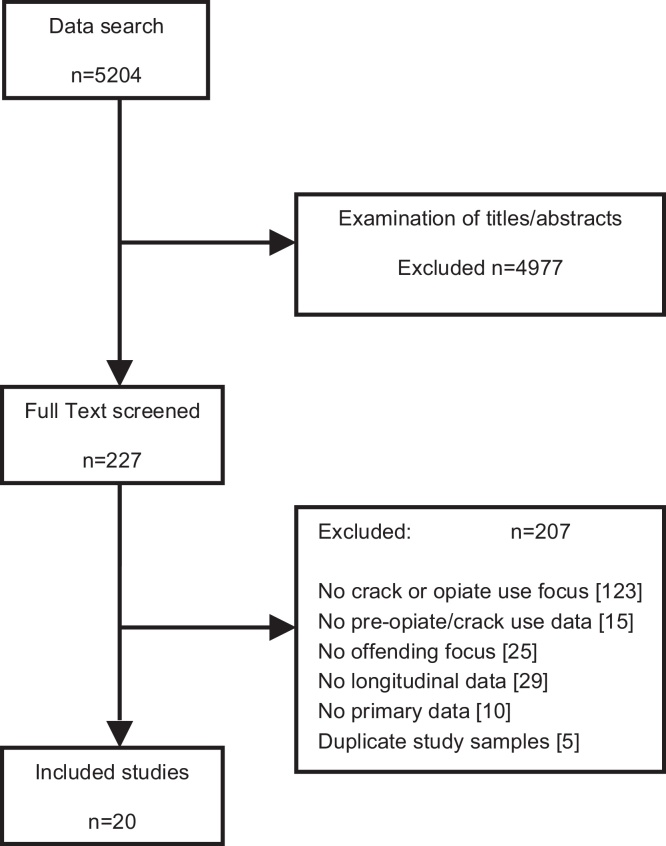
Review search: PRISMA flow diagram.

Screening of titles and abstracts excluded the majority, with 227 manuscripts proceeding to an examination of the full text. Most (*n* = 123, 54%) were excluded due to a lack of focus on opiates and/or crack use (see Fig. 1).

Six US studies (Anglin and Hser, 1987, Anglin and Speckart, 1988, Anglin et al., 1988a, Anglin et al., 1988b, Deschenes et al., 1991, Hser et al., 1987) identified by the search process utilised the same three samples of patients. We used data from one of these set of six studies (Anglin & Speckart, 1988) in our review. One included paper (Mott, 1975) provided a summary of studies reported elsewhere in greater detail (Mott and Rathod, 1976, Mott and Taylor, 1974) in addition to a summary of a third additional sample.

### Description of included studies

Twenty studies were included (see Table 1, Table 2), nine from the UK and 11 from the US. Research settings included drug treatment clinics (6), hospital (5), community (3), prison (3), not reported (1) and combined (2: hospital/general practice and prison/drug dependency clinic).

All studies focused on pathways through opiate use and crime. Nine studies with a focus on crack cocaine were excluded at the screening stage (no offending focus: 4; no longitudinal data: 4; no primary data: 1). Mean duration of addiction was reported by only four studies (range 12.9 months–16.8 yrs). The earliest data were collected on admission to a US hospital between 1935 and 1959 (O’Donnell, 1966); the most recent in 1987 (Jarvis & Parker, 1989).

Ten studies (50%) reported data from over 200 clients (range 34: Beckett & Lodge, 1971 to 765: Vorenberg & Lukoff, 1973, median 205, IQR 89–272). Studies were all male (6), all female (2), gender not reported (2) or mixed (10) with an average 70% (SD 15%) male. Ten studies reported mean age (29 yrs, range 20–37 yrs, 95% CI 25–32). Nine studies did not report ethnicity; elsewhere ethnic group was White, Black, Mexican American, Hispanic and Native American. Five studies (Gordon, 1973, James et al., 1979, Mott and Rathod, 1976, Parker and Newcombe, 1987, Cushman, 1974) reported use of a comparison group. Quality scores ranged from 3 to 7, out of a possible 9. Studies reporting offending rates, with the potential for pooling, had quality scores ranging from 5 to 7 (out of 9). Lower quality studies (scoring a total of 3 or 4) were characterised by inadequate details of follow-up and by a failure to control for the length of observation periods (Beckett and Lodge, 1971, Mott, 1975, Vorenberg and Lukoff, 1973).

### Findings of included studies

#### Age at opiate-use onset

Five studies report mean age at self-reported first opioid use (Table 3: range 16.6 yrs: Mott & Rathod, 1976 to 27.4 yrs: Chambers, Hinesley, & Moldestad, 1970; mean 19.6 yrs, 95% CI 17.4–21.8).

#### Age at offending onset

Five studies report mean age at (recorded) offending onset (Table 3: range 13.7 yrs: James et al., 1979 to 22.4 yrs: Chambers et al., 1970; mean 16.7 yrs, 95% CI 14.3–19.0). The CI (13.8–19.4) was widened by removal of one study (James & D’Orban, 1970) which reported mean age at first conviction rather than first arrest.

### Temporal order of opiate use and offending

**Fig. 2:**
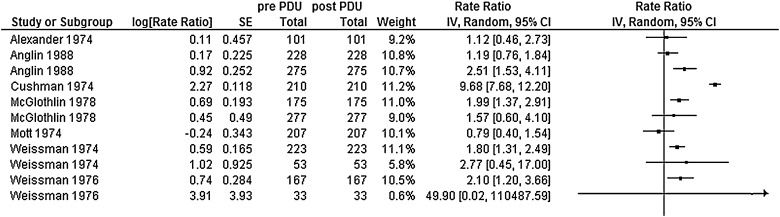
Rate ratios: total recorded offending. (All paired studies.)

Table 3, Table 4, Table 5 and Fig. 2 indicate that the mean age at (recorded) offending onset (16.7 yrs, 95% CI 14.3–19.0) precedes the mean age at opiate-use onset (19.6 yrs, 95% CI 17.4–21.8) by 2.9 yrs. Four studies (Anglin and Speckart, 1988, Chambers et al., 1970, James et al., 1979, McGlothlin et al., 1978) explicitly report mean age at opiate-use onset and at offending onset in the same sample (Table 3); however, all four fail to report the standard deviation for within-person delay from offending to opiate-use onset so therefore these estimates cannot be accurately pooled. Mean age at (recorded) offending onset preceded mean age at opiate-use onset in these four studies with the difference in means ranging from 2.6 yrs (Chambers et al., 1970) to 5.2 yrs (James et al., 1979).

### Type and volume of offending

Nine studies reported neither offending rates nor duration of observation periods and number of arrests/convictions to allow rate calculation. Ten (50%) reported offending rates (Alexander and McCaslin, 1974, Anglin and Speckart, 1988, Cushman, 1974, [Bibr bib0210][Bibr bib0260], Nurco and DuPont, 1977, Parker and Newcombe, 1987, Weissman et al., 1974, 1976; Wiepert, D’Orbán, & Bewley, 1979). Rates of serious crime increased after addiction onset in Nurco and DuPont (1977) but rates were presented in bands (e.g. 0.5–0.99) and could not be synthesised. Mott and Taylor (1974) did not present offending rates but reported number of convictions (males only) and median duration of each stage, enabling calculation of an approximate rate. Offence rates for the 10 studies with useable data are presented in Table 4, Table 5.

Data on the influence of onset-opiate use on total rates of offending (Table 4) were combined in a meta-analysis (Fig. 2), using a random effects model. Substantial heterogeneity (Chi^2^ = 689.9, df 14, *p* < 0.001, *I*^2^ = 98%) meant that it was uninformative to produce an overall rate ratio (RR). Using the vote method to synthesise data, RRs (comparing post-opiate use with pre-opiate use) ranged from 0.71 (95% CI 0.40–1.3: Alexander & McCaslin, 1974) to 25.7 (95% CI 8.9–74.0: Weissman, Marr, & Katsampes, 1976) across 27 subsamples from 10 studies. A positive association, i.e. higher rate post opiate onset, versus pre opiate onset, was observed in 22 of 27 subsamples. A negative association was seen in one subsample (Weissman et al., 1974) and four subsamples in two further studies reported equivocal associations, where CIs cross one (Alexander and McCaslin, 1974, Weissman et al., 1974) (Table 4). For the pooled data, 14 out of 15 independent samples had a positive association. We tested whether this was due to random error using the sign-test. The resulting *p* value was 0.001, demonstrating that this finding was unlikely to be due to chance.

Table 5 shows recorded offending rates for crime categories. RRs for theft, burglary and violence were derived from 7 studies; RRs for robbery from 5 studies. RRs for theft ranged from 0.63 (95% CI 0.38–1.0: Weissman et al., 1974) to 8.3 (95% CI 6.6–10.5: Cushman, 1974) with a positive association in 13 subsamples, an equivocal association in nine and a negative association in one. For burglary, RRs ranged from 0.74 (95% CI 0.45–1.2: Weissman et al., 1974) to 50.0 (95% CI 0.02–110727.0: Weissman et al., 1976) with positive associations in nine subsamples and equivocal associations in 13 (one subsample had a zero pre opiate onset rate). RRs for violence ranged from 0.39 (95% CI 0.11–1.4: Weissman et al., 1974) to 16.0 (95% CI 3.3–77.4: Weissman et al., 1974) with positive associations in six subsamples and equivocal associations in 15 (two subsamples had zero pre opiate onset rates). For robbery, RRs ranged from 0.50 (95% CI 0.18–1.4: McGlothlin et al., 1978) to 5.0 (95% CI 1.6–15.8: McGlothlin et al., 1978); only five subsamples had positive associations, 15 equivocal associations and one subsample had a zero pre opiate onset rate.

Drug offence rates were not pooled due to the expectation that these would increase post-opiate use onset. Two studies reported data on forgery (Anglin and Speckart, 1988, McGlothlin et al., 1978); both finding an increase post opiate onset but from zero pre opiate onset rates in one study. Rates of sexual assault were reported in three studies (Cushman, 1974, Mott and Taylor, 1974, Weissman et al., 1976); one had zero rates (Weissman et al., 1976) and two were unable to detect a reliable change (Cushman, 1974, Mott and Taylor, 1974). Rates of prostitution were reported in three studies (Cushman, 1974, Weissman et al., 1974; 1976) two with all-female subsamples (see Table 1, Table 5). Rates decreased in one group (RR 0.69, CI 0.35–1.3) and increased in the other from a previously zero rate.

Table 5 suggests offending rates increase post-opiate use onset across the crime categories of theft and burglary (Supplementary Figs. 2 and 3). Funnel plots of effect estimate (RR) against standard error (log RR) show potential publication bias with a lack of non-significant findings from studies with small sample sizes.

Meta-regression was used to examine potential sources of between-study heterogeneity including: sample size, study quality score, country, setting, study date and gender; 95% CIs for adjusted RRs all crossed one with no significant predictors of the Rate Ratio.

### Studies comparing offending with a non-drug-using population

Although not an inclusion criterion, those studies which attempted to set offending details against a comparison group were examined further. Cushman (1974) compared arrest rates for an addict group (*n* = 210) with the general population in the study location (*n* = 191,000). Property crime arrest rates were comparable pre opiate onset (0.9 vs. 0.7) but higher post opiate onset in the addict group (8.9 vs. 0.7). However, comparison group data were derived from arrest rates divided by the total population and age was not accounted for. Parker and Newcombe (1987) used a comparison group (*n* = 188) of non-drug-using offenders. User offenders’ acquisitive crime rates increased substantially (0.3–1.2) compared to non-drug-using offenders (0.2–0.7). However, analysis assumed the timing of opiate-use onset based on that of a local heroin epidemic (Parker, Bakx, & Newcombe, 1986) and did not adjust for age, although the groups’ age distributions were similar (17–32 yrs). James et al. (1979) compared an opiate-using addict group (*n* = 68) with a non-addict offender group (*n* = 64). Self-reported mean age of first criminal involvement was lower for addicts (13.0 vs. 14.2 yrs) although the standard error for the difference in means was not reported. As adults, addicts were more likely to commit forgery (21% vs. 8%) although no details are provided on pre-opiate use offence volume and type. Gordon (1973) analysed findings according to heroin vs. other drug use. Pre-opiate onset conviction levels were similar; the incidence of larceny increased post-opiate onset (37–80%) for the heroin user group but not the group using other drugs. However, offence rates and details of observation periods were not presented.

### Potential interactions between opiate use and offending

Review findings were used to examine how epoch, gender, volume and type of pre opiate onset offending and age at opiate use onset might impact upon the opiate use–crime relationship. Ethnicity was not included due to differences between countries and epochs and the confounding effects of socio-economic factors (Galea et al., 2004, Lillie-Blanton et al., 1993).

Four studies >(Cushman, 1974, McGlothlin et al., 1978, Nurco and DuPont, 1977, O’Donnell, 1966) suggest later cohorts have higher arrest rates both pre- and post-opiate use onset. Cushman (1974) suggested that increases in the extent of the relationship between opiate use and offending over time (start of daily use plotted against arrest frequency in the same jurisdiction) result from an increase in the price of heroin and increased law enforcement efforts.

Where reported, studies suggest a gender impact on the opiate use–crime relationship with greater escalation and a somewhat different pattern of offending in females. Substantial increases occur post-opiate use onset for females (e.g. pre-addiction larceny rate of 0.15 for males vs. 0.05 for females, increasing to 0.35 post-addiction in both: Weissman et al., 1976). Increases were also more likely for burglary (50-fold increase with a wide CI from 0.002 to 0.10, 95% CI 0.02–110727.0 in females, compared to a 2-fold increase from 0.11 to 0.23 in males: Weissman et al., 1976) and assault (16-fold increase from 0.03 to 0.50 (95% CI 3.4–82.5) in females compared to a small increase from 0.10 to 0.11 in males: Weissman et al., 1974). Wiepert et al. (1979) report that conviction rates did not differ significantly by gender.

Disaggregating by crime type, studies suggest participation in acquisitive crime pre- and post-opiate use onset, with post-onset escalation mainly limited to acquisitive crime (Jarvis and Parker, 1989, Parker and Newcombe, 1987). Minor acquisitive crime, e.g. theft, appears more likely to precede addiction (Anglin & Speckart, 1988). The proportion involved in property crime increases post-opiate use onset (Weissman et al., 1974, 1976; Wiepert et al., 1979), e.g. from below 50% to over 80% in Anglin and Speckart (1988). A number of studies suggest no significant impact of opiate-use onset on violent offending (Alexander and McCaslin, 1974, Mott and Taylor, 1974, Vorenberg and Lukoff, 1973) while others do (Mott and Rathod, 1976, O’Donnell, 1966).

Findings from studies that group individuals on the basis of age at opiate-use onset (O’Donnell, 1966, Vorenberg and Lukoff, 1973, Weissman et al., 1974, 1976) suggest this affects the opiate use–crime relationship; large increases in offending post-opiate onset are observed in early-onset cases with a history of arrests prior to drug use (two studies recruit at prison entry: Weissman et al., 1974, 1976). The youngest opiate use onset group is characterised by increases in assault (2-fold increase from 0.10 to 0.19: 13–20 onset: Weissman et al., 1974) and larceny (7-fold increase from 0.05 to 0.35: 13–17 onset: Weissman et al., 1976), whereas later opiate use onset groups had greater increases for burglary and robbery (5-fold increase for burglary from 0.12 to 0.59: 21–25 onset: Weissman et al., 1974).

### Other findings

The impact of money spent on drugs on crime participation was highlighted (Anglin and Speckart, 1988, Cushman, 1974, Gordon, 1973), although participants were described as unable to provide reliable estimates of drug expenditure (James et al., 1979). Similarly, income derived from crime was reported (James et al., 1979, McGlothlin et al., 1978) although one study cautioned that participants did not consider the value of goods stolen for personal use rather than resale as illegal income, leading to substantial underestimation (James et al., 1979).

Studies commented on the intervening role played by (undetected) drug dealing in determining the extent of the drugs-crime relationship (Anglin and Speckart, 1988, James et al., 1979). Over 50% of participants’ time was taken up by dealing drugs (McGlothlin et al., 1978), which could contribute two-thirds of addicts’ income (James et al., 1979). Self-report confirms an increase in acquisitive crime; 65% of heroin users (*n* = 46) in one study reported supporting their drug use via acquisitive crime (Jarvis & Parker, 1989). A further study recorded participants’ perceptions of addiction onset on crime; 84% reported no change in assault, whereas 46% reported a substantial increase in theft (Weissman et al., 1976).

## Discussion

The literature on pathways through opiate/crack use and offending was systematically reviewed. This appears to be the first systematic review to concentrate specifically on such pathways. Twenty studies (11 US, 9 UK) were included from over 5000 hits. Most took place in drug treatment clinics and all focused on opiate use.

### Summary of main findings

Mean age at opiate-use onset was 19.6 yrs and mean age at offending onset (usually arrest) was 16.7 yrs, pointing to evidence supporting the pathway whereby offending precedes opiate use initiation in studies setting out sufficient data on the temporal sequence of opiate use and offending. Individual studies suggest increases in rates of acquisitive crime, such as theft (Jarvis & Parker, 1989), burglary (O’Donnell, 1966), and robbery (Weissman et al., 1976) following opiate-use onset. Substantial between-study heterogeneity (above 80%), not explained by meta-regression, meant that rate ratios should not be pooled via meta-analysis. Studies reporting crime rates suggest that these increase following opiate-use onset: positive association in 22 subsamples, equivocal association in four subsamples and a negative association in one subsample (RR range 0.71–25.7). The majority of independent samples (14/15) had a positive association; this finding is unlikely to be due to chance, according to a sign test (*p* = 0.001). For individual crime categories, the strongest evidence was available for theft (positive association in 13 subsamples, equivocal association in 9 subsamples and a negative association in one: RR 0.63–8.3). Gender and age at opiate-use onset appear to moderate the strength of the opiate use–crime relationship. Opiate-use onset escalates already-existing criminal behaviour, particularly for acquisitive crime types, although the available evidence is highly heterogeneous and not up to date.

### Limitations

None of the included studies reported on the use of crack cocaine; findings therefore relate to pathways through opiate use and crime. In England, opiate users account for 79% of the drug treatment population, meaning that findings have relevance to the UK situation (PHE, 2014). Although heroin use would appear to be declining in the UK, given low rates of use among young people in available indicator data (2013/14 NDTMS data), there remains an older population of heroin users who are targeted for intervention on the premise that their drug use causes crime. The focus on opiate use is also important given recent concern over increasing mortality rates associated with opiate use in both the UK (Pierce, Bird, Hickman, & Millar, 2015a) and other countries, such as Australia (Kimber, Larney, Hickman, Randall, & Degenhardt, 2015) and the US (Jones, 2013). Available evidence does point to divergent patterns of problematic drug use between countries. For example, methamphetamine use is expanding across South East Asia (UNODC, 2015) and is the focus of interventions to target drug-related crime in the US (Hayhurst et al., 2015), potentially limiting the generalisability of review findings. The recent increase in heroin use in the US (Cicero et al., 2014, Jones, 2013), however, does indicate the potential for further work on the nuances of the opiate-crime relationship in a contemporary population.

The absence of studies focusing on crack cocaine in the review highlights that much of the available evidence base in this area predates the widespread prevalence of crack cocaine use. In addition, particularly in the UK, crack is a substance that opiate users have added to their drug-taking repertoire, meaning that crack use in the absence of opiate use is relatively uncommon in the UK (PHE, 2014).

None of the studies compared crime rates between opiate users and non-drug-users to establish whether opiate-use onset is associated with surplus crime, while controlling for confounding factors, such as age or incarceration. In essence, included studies use a mirror-image design of before/after opiate-use onset in the same person. Most research in this field does not control for age-confounding: there is typically a steep rise in offending through adolescence (Sweeten, Piquero, & Steinberg, 2013), the typical period for opiate-use onset (Lynskey & Hall, 1998). Thus, without a comparison group, it is not possible to ascribe opiate-use onset as the cause of increased offending. A few studies attempted to set offending details against a comparison group but these were characterised by design flaws, such as not controlling for age (Cushman, 1974, Parker and Newcombe, 1987), providing insufficient details on pre-opiate use offending (James et al., 1979) and not taking account of differential observation periods (Gordon, 1973).

All studies originated from the UK and US, despite a comprehensive literature search. The available evidence base may be biased due to the absence of published studies with non-significant results (Vecchi, Belleudi, Amato, Davoli, & Perucci, 2009); the funnel plot pointed to this possibility. Eligibility was restricted to peer-reviewed evidence to ensure a degree of robustness; previous work points to often important differences between, for example, conference abstracts and peer-reviewed manuscripts reporting the same study (Toma et al., 2006). Only two studies used baseline data collected after 1980 (Jarvis and Parker, 1989, Parker and Newcombe, 1987) and are, therefore, better able to inform the UK situation subsequent to the heroin ‘epidemics’ of the early-1980s (Parker, Newcombe, & Bakx, 1987). The lack of contemporary research in the available literature suggests that researchers consider that the opiate use–crime relationship is completely determined. However, this review highlights that data essential to establishing the temporal nature of the relationship have not previously been synthesised. In addition, the review highlights the dearth of studies covering the period prior to drug-use initiation and the lack of high-quality research, employing a non-drug-using comparison group, needed to comprehensively implicate onset-opiate use in the acceleration of existing offending behaviour.

Differences in epoch are a conspicuous limitation in extrapolating review findings to current opiate-using offenders. Earlier cohorts may differ in critical respects: they are more likely to (1) have become addicted (and sustained their addiction) via prescribed treatment (Chambers et al., 1970); (2) be older at addiction onset (O’Donnell, 1966); and (3) engage in behaviour indicative of deviant rather than criminal tendencies (Beckett and Lodge, 1971, O’Donnell, 1966). Difficulties also arise when accounting for findings from UK studies (Beckett and Lodge, 1971, Wiepert et al., 1979) undertaken when heroin was available on prescription. This highlights that the drugs-crime link will vary across both time and geography (Seddon, 2000); given that it is not an invariant relationship, but is shaped by the wider context, a review drawing on studies from diverse locations and time periods will, understandably, be characterised by between-study heterogeneity. However, heterogeneity may have been exaggerated by post hoc selective sub-grouping of study participants.

We agree with others that large longitudinal datasets are necessary to investigate developmental causality in offending (Blumstein & Cohen, 1987). We therefore focused on longitudinal data with corroborative official crime records. Much of the existing literature derives from cross-sectional studies of self-report data (e.g., Klee and Morris, 1994, Swan and Goodman-Delahunty, 2013), the accuracy of which will be tempered by recall bias, particularly over long time periods (Coughlin, 1990). However, recorded crime underestimates levels of criminal activity; there will be a lag between first crime and first arrest and between first arrest and first conviction. Self-reported mean age at first crime precedes age at first juvenile arrest by 1.4 yrs in one included study (James et al., 1979). It is tenable that studies suggesting that drug use precedes crime include participants whose offending went undetected prior to drug use, indicating the need for self-report data to supplement information on recorded sanctioned offending.

Not all studies explicitly supplied information about offending rates. Even where rates were provided, studies with shorter post-opiate use onset follow-up will have higher RRs due to the relationship between age and crime; analyses stratified by age at cohort entry, e.g. pre-specified 5-year age epochs (15–19, 20–24, etc.) are more appropriate. As a minimum, studies in this field would have greater value if the following were consistently reported: Criminal Justice System (CJS) events pre-opiate use onset; person-years (PYs) pre-opiate use onset; mean age at opiate use onset with standard deviation (sd); CJS events post-opiate use onset; PYs post-opiate use onset; and mean years of follow-up (sd) post-opiate use onset. For before/after studies, the SD for within-client delay from CJS-onset to opiate use onset is required in order to accurately pool estimates.

Meta-analysis was used to pool rate ratios (RR) of post-drug use initiation to pre-drug use initiation. An issue with this measure is that the available data were paired, i.e. the comparison between pre-opiate use and post-opiate use onset was undertaken on the same individual; subject-specific effects will mean that the two periods will be highly correlated. All included studies surveyed failed to account for these effects, therefore the estimates will be overly precise. Similarly, a number of subsamples were derived from two studies by Weissman (Weissman et al., 1974, 1976) leading to the potential for unobserved heterogeneity. Insufficient data were available to examine the impact of socioeconomic status on between-study heterogeneity; this factor has been linked to both crime and drug use (Gauffin et al., 2013). Substantial levels of between-study heterogeneity meant that data were unable to be pooled and were presented using the vote method. The vote method is limited as non-significant findings may be due to a lack of statistical power rather than the absence of an effect (Borenstein et al., 2009, Greenland and O’Rourke, 2008).

### Implications and findings in relation to other evidence

#### Temporal order of opiate use and offending

The mean age of recorded offending preceded the mean age at opiate-use onset by 2.9 yrs. This can be compared with UK norms reported by Pudney (2002) comprising survey data on 12–30 yr olds. Mean age of onset for minor crime (14.5 yrs) in the Pudney sample preceded mean age at opiate-use onset by 3 years for heroin and 3.9 years for crack cocaine. In the studies reviewed here, offending onset preceded opiate-use onset, with rates of total offending increasing post-opiate use. Notwithstanding the limitations imposed by design flaws in the included studies, the implication is that opiate-use onset escalates already-existing criminal behaviour.

#### Type and volume of offending

Drugs offences were not synthesised as increased rates were expected post-opiate use onset. RRs for other offence types suggest that offending increases post-opiate use, particularly for theft and possibly burglary. As concluded by one of the studies, opiate users focus on those offence types which provide a satisfactory return, are within the skill set of the individual and carry the lowest risk of arrest (James et al., 1979).

Findings for violent offending were less certain. Previous work suggests no relationship between heroin onset and onset or escalation of violent crime (Parker & Auerhahn, 1998). The association of non-opiate drugs, such as crack cocaine, with violent crime may be due to usage in combination with alcohol (Martin, Maxwell, White, & Zhang, 2004). However, others have highlighted alcohol's facilitative role in acts of crime by heroin users (Strug et al., 1984) suggesting that the role of alcohol in the drugs-crime link requires further investigation.

Similarly, opiate users are often poly-drug users, limiting capacity to ascribe causality specifically to opiates. For example, the opiate-using sample in one study reviewed here (Gordon, 1973) also used amphetamines, a drug associated with both violent (Wright & Klee, 2001) and non-violent crime (Klee & Morris, 1994). In terms of contemporary samples, 40% of opiate users treated during 2012/13 in England also used crack (PHE, 2013). Others have highlighted that the number of drug types used correlates with offending rates (Bennett & Holloway, 2005) and that heroin users, for example, commit different types of crime according to their non-opiate adjunctive drug use (Shaffer, Nurco, Ball, & Kinlock, 1985). Further robust research on the nature of offending in poly-drug users is required.

#### Interactions with opiate use and offending

Females have lower offence rates pre-opiate use than males, confirming previous findings (Hall et al., 1993) but a greater escalation of criminal behaviour post-opiate use. As females’ post-opiate use crime rates escalate from lower levels, higher RRs result. Others have argued that female opiate using offenders develop more serious drug dependence than their male counterparts (Holloway & Bennett, 2007). Large increases in post-opiate use crime are observed in early-onset opiate use preceded by delinquency and crime, although in the absence of a control group it is impossible to rule out an age effect operating here and it is not known whether sub-grouping by age at opiate use onset took place post hoc.

Differences may exist between opiate user and non-opiate user samples prior to opiate-use onset, thus research in this field should employ a matched non-opiate user comparison group. For example, it is suggested that opiate users have high levels of criminal behaviour prior to onset-opiate use with the level of this predicting escalation post-opiate use onset (Shaffer et al., 1987).

## Conclusions

Given the prominence of the drugs-crime link in drug policy, there is a surprising lack of robust evidence focusing specifically on pathways through opiate use and offending. We have established that the evidence base is: (a) out of date and may not apply to the current situation; (b) methodologically very weak. Understanding the temporal nature of the relationship between opiate use and crime is of prime importance to the development of strategies designed to intervene at critical points during the natural history of drug use and offending. We therefore recommend that, if policy continues to be based on the assumed link between opiate use and crime, there is a need for new, methodologically more appropriate research considering the influences on crime. This needs to utilise a longitudinal design with a matched non-opiate user comparison group.

## Figures and Tables

**Table 1:** Characteristics of 20 included studies.

Study	Country	Drug use focus	Setting	Sample size	Gender	Age^a^
Alexander and McCaslin (1974)	US	Heroin	Outpatient methadone treatment	*N* = 101/160	Male 65% of *N* = 160	Mean 24 yrs (*N* = 160)
Anglin and Speckart (1988)	US	Narcotics (opiates)	Methadone clinics	*N* = 503/671	Male 100%	Mean 20.5 yrs (at addiction)
Beckett and Lodge (1971)	UK	Heroin	Hospital addiction unit	*N* = 34	Male 100%	Mean 20 yrs (at addiction)
Chambers et al. (1970)	US	Narcotics (opiates)	Hospital narcotic treatment facility	*N* = 168	Female 100%	Mean 34 yrs (at admission)
Cushman (1974)	US	Narcotics (opiates)	Methadone clinic	*N* = 210/269	Female 22%	Mean 33 yrs (at admission)/range 18–77 yrs
Gordon (1973)	UK	Narcotics (heroin)	Drug clinic	*N* = 60 (at baseline)	Male 100% of *N* = 60 (baseline)	Mean 22 yrs (SD 3.5 yrs)
James and D’Orban (1970)	UK	Heroin	Remand centres & women's prison	*N* = 116	Male 43%	Mean 24 yrs (M): 90% <30 yrs Mean 20 yrs (F): range 16–50 yrs
James et al. (1979)	US	Opiates	Not reported	*N* = 134/268	Female 100%	Mean 25 yrs (*N* = 268)
Jarvis and Parker (1989)	UK	Heroin	Prisons & Drug Dependency Units	*N* = 46	Male 63%	Mean not reported 91% < 29 yrs
McGlothlin et al. (1978)	US	Narcotics (opiates)	Addict program	*N* = 452/690	Male 100%	Mean 25 yrs (at admission)
Mott and Taylor (1974)	UK	Opiates	Psychiatric Hospitals & General Practice	*N* = 273	Male 76%	Mean not reported
Mott (1975)	UK	Opiates	Hospital	*N* = 74/99	Male 100%	Mean not reported 56% 14–20 yrs (when notified to Home Office)
Mott and Rathod (1976)	UK	Heroin	Community	*N* = 80	Male 84%	Mean not reported
Nurco and DuPont (1977)	US	Narcotics (opiates)	Community	*N* = 252	Male 100%	Mean not reported
O’Donnell (1966)	US	Narcotics b	Hospital narcotic treatment facility	*N* = 266	Male 80%	Mean not reported
Parker and Newcombe (1987)	UK	Heroin	Community	*N* = 91/279	Not reported	Mean “about 21” (total study): 93% 17–32 yrs
Vorenberg and Lukoff (1973)	US	Heroin	Addiction treatment facility	*N* = 765	Not reported	Mean not reported
Weissman et al. (1974)	US	Opiates	Jail	*N* = 276/282	Male 81%	Median 26 yrs
Weissman et al. (1976)	US	Opiates	Jail	*N* = 200	Male 84%	Mean 27 yrs/range 19–56 yrs
Wiepert et al. (1979)	UK	Opiates	Hospital drug dependence clinics	*N* = 236	Male 50%	Mean not reported

a SD not reported in the majority of studies

b presumed opiates based on publications using the same sample source

**Table 2:** Study design features and assessment of study quality.

	Data collection	Nature of data	Observation periods*	Study quality			
Study				Selection^a^ (max = 4)	Comparability^b^ (max = 2)	Outcome^c^ (max = 3)	Total score (max = 9)
Alexander 1974	Retrospective	Objective: CR	Not specified	2	1	3	6
Anglin and Speckart (1988)	Retrospective	Objective: CR Subjective: I	Pre Mean = 4.8 yrs Post Mean = 14.75 yrs	2	1	3	6
Beckett and Lodge (1971)	Unclear	Objective: court records Subjective: I	Not specified	1	0	2	3
Chambers et al. (1970)	Retrospective	Objective: CR Subjective: I	Pre Mean = 4.1 yrs Post Mean = 7.8 yrs	2	0	3	5
Cushman (1974)	Retrospective	Objective: CR	Pre Mean = 5.5 yrs Post Mean = 13.3 yrs	3	1	3	7
Gordon (1973)	Retrospective#	Objective: CR Subjective: I	Pre Mean = 60.5 m Post not specified	3	1	3	7
James and D’Orban (1970)	Retrospective	Objective: CR	Not specified	2	0	3	5
James et al. (1979)	Retrospective	Objective: CR Subjective: I	Pre > 5 yrs Post Mean = 1.8 yrs	3	1	3	7
Jarvis and Parker (1989)	Retrospective	Objective: CR Subjective: SR	Not specified	2	1	2	5
McGlothlin et al. (1978)	Retrospective	Objective: CR Subjective: SR	Pre = 12 m Post = 12 m	2	1	3	6
Mott and Taylor (1974)	Retrospective	Objective: CR	Pre Mean = 8.4 yrs Post Mean = 2.8 yrs	2	0	3	5
Mott (1975)	Retrospective	Objective: CR	Pre not specified Post =2 yrs	2	0	2	4
Mott and Rathod (1976)	Retrospective	Objective: CR Subjective: I	Pre not specified Post = 2–4 yrs	2	0	3	5
Nurco and DuPont (1977)	Retrospective	Objective: CR Subjective: I	Not specified	2	1	3	6
O’Donnell (1966)	Retrospective	Objective: CR	Pre not specified Post = 2–28 yrs	2	0	3	5
Parker and Newcombe (1987)	Retrospective	Objective: CR	Pre not specified Post = 1–9 yrs	3	1	3	7
Vorenberg and Lukoff (1973)	Retrospective	Objective: CR	Not specified	2	0	1	3
Weissman et al. (1974)	Retrospective	Objective: CR	Pre = 1.5–9.6 yrs Post = 3.3–4.3 yrs	2	1	3	6
Weissman et al. (1976)	Retrospective	Objective: CR Subjective: I	Pre = 2.3–7.5 yrs Post = 3.4–5.8 yrs	2	1	3	6
Wiepert et al. (1979)	Retrospective	Objective: CR	Pre = max 7.1 yrs Post = max 8.5 yrs	2	1	3	6

a Representativeness, selection, exposure.

b Study controls.

c Assessment of outcome, length of FU, adequate FU. CR: Criminal Records. I: Interview. SR: self-report.

* Estimated from data presented in the paper.

# FU reported in other publications by the same author.

**Table 3:** Age at opiate use onset and offending onset.^a^

Study (lead author)	Mean age at offending onset	Other age/offending data	Mean age at opiate use onset	Other age/drug data
Alexander (1974)	–	Takes 17 yrs as the start point for crime classification	–	Takes 17 yrs as the start of the pre-addiction period
Anglin 1988	16.2 yrs/15.2 yrs (self-report mean age first arrest)	–	19.2 yrs/18.6 yrs	Mean age at addiction = 20.7 yrs/20.3 yrs
Beckett (1971)	–	–	–	Mean age at addiction = 20 yrs
Chambers (1970)	22.4 yrs/21.2 yrs (mean age first arrest)	–	27.4 yrs/21.3 yrs	–
Cushman (1974)	–	Takes 15 yrs as the start point for crime classification	–	Takes 15 yrs as the start of the pre-addiction period Mean age at addiction = 20.5 yrs
Gordon (1973)	–	–	–	–
James (1970)	17.2 yrs (mean age first conviction) (M)	–	–	50% opiate use onset at 18–19 yrs (F) Mean age first drug offence = 22.8 yrs (M)
James (1979)	14.3 yrs/13.7 yrs (mean age first juvenile arrest)	12.98 yrs/12.22 yrs (self-report mean age first criminal involvement)	20.1 yrs/18.3 yrs	–
Jarvis (1989)	–	–	–	–
McGlothlin (1978)	15.1 yrs/14.8 yrs (mean age first arrest)	–	18.6 yrs/19 yrs	Mean age at addiction = 20.4 yrs/20.9 yrs
Mott 1974	–	Age first conviction: 8–14 yrs (15%)/14–17 yrs (20%)/17–21 yrs (25%)	–	19.6 yrs = median age at start of “early opiate use”
Mott (1975)	–	–	–	–
Mott (1976)	–	Males convicted <21 yrs (35%) Females court appearance <17 yrs (38.5%)	16.6 yrs/17 yrs	–
Nurco (1977)	–	–	–	Takes 14 yrs as the start of the pre- opiate use period
O’Donnell (1966)	–	–	–	Median age at addiction= 31.3 yrs (M) 30 yrs (F)
Parker (1987)	–	Analysed data by age at onset offending <16 yrs vs. >16 yrs	–	Assumes age of onset = 16 yrs (from previous work: Parker et al., 1986)
Vorenberg (1973)	–	–	–	Mean age at addiction = 21 yrs
Weissman (1974)	–	–	–	Majority (39%) 13–20 yrs (assumes age at first drug arrest as onset age)
Weissman (1976)	–	–	–	41% 18–21 yrs at addiction (daily use)
Wiepert (1979)	–	Males convicted < 17.3 yrs (32%) Females convicted < 16.7 yrs (12%)	–	–
*Mean (95% CI)*	*16.7* yrs *(14.3–19.0)*		19.6 yrs (17.4–21.8)	

a None of the before/after studies reported a within-client SD for delay (opiate use onset-CJS onset).

**Table 4:** Total recorded offending.

	Pre-opiate use offending rate	Post-opiate use offending rate	Rate ratio	95% CI^a^
*Conviction rates*				
Alexander 1974 (*n* = 101)	0.28	0.2	0.71	0.40–1.3
Jarvis (1989) (*n* = 46)	1.0	2.2	2.2	1.5–3.1
Mott 1974 (male group: *n* = 207)	0.46	0.63	1.4	1.1–1.7
Parker (1987) (*n* = 91)^d^	0.26	1.2	4.6	3.8–5.6
Parker (1987) (*n* = 91)^e^	0.22	0.42	1.9	1.5–2.4
Wiepert (1979)^h^ (all: *n* = 236)^f^	0.08	0.50	6.2	5.3–7.3
Wiepert (1979)^h^ (all: *n* = 236)^g^	0.08	0.38	4.7	4.0–5.6
Wiepert (1979)^h^ (male group: *n* = 119)^f^	0.12	0.64	5.3	4.4–6.5
Wiepert (1979)^h^ (male group: *n* = 119)^g^	0.12	0.48	4.0	3.3–4.9
Wiepert (1979)^h^ (female group: *n* = 117)^f^	0.04	0.35	8.7	6.6–11.7
Wiepert (1979)^h^ (female group: *n* = 117)^g^	0.04	0.27	6.7	5.0–9.1

*Arrest rates*				
Alexander 1974 (*n* = 101)	0.55	0.42	0.76	0.51–1.1
Anglin 1988 (White group: *n* = 275)	0.81	1.2	1.5	1.3–1.8
Anglin 1988 (Mexican American group: *n* = 228)	0.99	1.4	1.4	1.2–1.7
Cushman, 1974 (*n* = 210)	0.03	0.35	11.3	9.0–14.3
McGlothlin (1978) (1962–64 group: *n* = 277)	1.4	1.9	1.4	1.2–1.6
McGlothlin (1978) (1970 group: *n* = 175)	1.7	2.7	1.6	1.4–1.8
Weissman (1974) (Black group: *n* = 113)^c^	1.2	1.6	1.3	1.0–1.6
Weissman (1974) (Hispanic group: *n* = 86)^c^	1.6	1.6	1.0	0.79–1.3
Weissman (1974) (White group: *n* = 77)^c^	1.3	1.5	1.1	0.88–1.5
Weissman (1974) (male group: *n* = 223)^c^	1.4	1.7	1.2	1.0–1.4
Weissman (1974) (female group: *n* = 53)^c^	1.3	2.1	1.6	1.2–2.1
Weissman (1974) (13–20 onset group: *n* = 109)^c^	1.7	1.4	0.80	0.64–0.99
Weissman (1974) (21–25 onset group: *n* = 99)^c^	1.2	2.6	2.1	1.7–2.7
Weissman (1974) (26+ onset group: *n* = 68)^c^	1.1	1.2	1.1	0.82–1.5
Weissman (1976) (Black group: *n* = 73)^b^	0.56	2.8	4.9	3.5–6.9
Weissman (1976) (Black group: *n* = 73)^c^	0.53	1.2	2.3	1.6–3.4
Weissman (1976) (Mexican American group: *n* = 81)^b^	0.46	2.3	5.0	3.5–7.1
Weissman (1976) (Mexican American group: *n* = 81)^c^	0.40	0.93	2.3	1.5–3.5
Weissman (1976) (White group: *n* = 46)^b^	0.23	1.7	7.6	4.0–14.4
Weissman (1976) (White group *n* = 46)^c^	0.22	0.91	4.1	2.1–8.2
Weissman (1976) (male group: *n* = 167)^b^	0.49	2.4	4.9	3.9–6.2
Weissman (1976) (male group: *n* = 167)^c^	0.45	1.1	2.4	1.8–3.1
Weissman (1976) (female group: *n* = 33)^b^	0.18	2.1	11.4	4.9–26.5
Weissman (1976) (female group: *n* = 33)^c^	0.17	0.97	5.7	2.3–14.0
Weissman (1976) (13–17 onset group: *n* = 51)^b^	0.07	1.8	25.7	8.9–74.0
Weissman (1976) (13–17 onset group: *n* = 51)^c^	0.07	0.94	13.4	4.6–39.4
Weissman (1976) (18–21 onset group: *n* = 83)^b^	0.57	2.4	4.2	3.1–5.8
Weissman (1976) (18–21 onset group: *n* = 83)^c^	0.54	1.1	2.1	1.4–2.9
Weissman (1976) (22+ onset group: *n* = 66)^b^	0.54	2.7	5.0	3.5–7.2
Weissman (1976) (22+ onset group: *n* = 66) c	0.48	1.1	2.2	1.4–3.4

a Calculated using estimated number of events if not reported, mean SE where missing.

b Total reported crime.

c Total non-drug reported crime.

d Total acquisitive crime (burglary, theft).

e Total non-acquisitive crime.

f Total convictions.

g Total non-drug offence convictions.

**Table 5:** Recorded crime categories.

	Pre-opiate use offending rate	Post-opiate use offending rate	Rate ratio	95% CI^a^
*Theft*				
Alexander 1974 (*n* = 101)	0.09	0.06	0.67	0.24–1.9
Anglin 1988 (White group: *n* = 275)	0.03	0.11	3.7	1.7–7.9
Anglin 1988 (Mexican American group: *n* = 228)	0.04	0.10	2.5	1.2–5.4
Cushman, 1974 (*n* = 210)^b^	0.01	0.11	8.3	6.6–10.5
McGlothlin (1978) (1962–64 group: *n* = 277)^c^	0.05	0.10	2.0	1.0–3.8
McGlothlin (1978) (1970 group: *n* = 175)^c^	0.12	0.23	1.9	1.1–3.2
Mott 1974 (male group: *n* = 207)	0.15	0.14	0.95	0.58–1.5
Weissman (1974) (Black group: *n* = 113)	0.47	0.55	1.1	0.80–1.7
Weissman (1974) (Hispanic group: *n* = 86)	0.41	0.58	1.4	0.92–2.2
Weissman (1974) (White group: *n* = 77)	0.50	0.31	0.63	0.38–1.0
Weissman (1974) (male group: *n* = 223)	0.43	0.50	1.2	0.88–1.5
Weissman (1974) (female group: *n* = 53)	0.59	0.86	1.5	0.93–2.3
Weissman (1974) (13–20 onset group: *n* = 109)	0.52	0.49	0.95	0.65–1.4
Weissman (1974) (21–25 onset group: *n* = 99)	0.41	0.75	1.8	1.2–2.7
Weissman (1974) (26+ onset group: *n* = 68)	0.44	0.41	0.94	0.56–1.6
Weissman (1976) (Black group: *n* = 73)	0.20	0.50	2.5	1.4–4.6
Weissman (1976) (Mexican American group: *n* = 81)	0.11	0.27	2.4	1.1–5.3
Weissman (1976) (White group: *n* = 46)	0.08	0.25	3.1	0.97–10.1
Weissman (1976) (male group: *n* = 167)	0.15	0.35	2.3	1.5–3.7
Weissman (1976) (female group: *n* = 33)	0.05	0.35	7.0	1.4–35.7
Weissman (1976) (13–17 onset group: *n* = 51)	0.05	0.35	7.0	1.9–26.0
Weissman (1976) (18–21 onset group: *n* = 83)	0.19	0.35	1.8	1.0–3.4
Weissman (1976) (22+ onset group: *n* = 66)	0.14	0.36	2.6	1.2–5.5

*Burglary*				
Alexander 1974 (*n* = 101)^d^	0.09	0.10	1.1	0.45–2.7
Anglin 1988 (White group: *n* = 275)	0.08	0.20	2.5	1.5–4.1
Anglin 1988 (Mexican American group: *n* = 228)	0.16	0.19	1.2	0.76–1.8
Cushman, 1974 (*n* = 210)^e^	0.01	0.09	9.7	7.7–12.2
McGlothlin (1978) (1962–64 group: *n* = 277)	0.16	0.25	1.6	0.60–4.1
McGlothlin (1978) (1970 group: *n* = 175)	0.23	0.46	2.0	1.4–2.9
Mott 1974 (male group: *n* = 207)^g^	0.09	0.07	0.79	0.40–1.5
Weissman (1974) (Black group: *n* = 113)	0.12	0.47	4.0	2.2–7.3
Weissman (1974) (Hispanic group: *n* = 86)	0.32	0.26	0.83	0.48–1.4
Weissman (1974) (White group: *n* = 77)	0.23	0.24	1.0	0.54–2.0
Weissman (1974) (male group: *n* = 223)	0.25	0.46	1.8	1.3–2.5
Weissman (1974) (female group: *n* = 53)	0.03	0.08	2.8	0.45–17.0
Weissman (1974) (13–20 onset group: *n* = 109)	0.34	0.26	0.74	0.45–1.2
Weissman (1974) (21–25 onset group: *n* = 99)	0.12	0.59	4.8	2.6–8.9
Weissman (1974) (26+ onset group: *n* = 68)	0.13	0.30	2.3	1.1–5.0
Weissman (1976) (Black group: *n* = 73)	0.10	0.18	1.8	0.73–4.4
Weissman (1976) (Mexican American group: *n* = 81)	0.13	0.24	1.8	0.9–3.9
Weissman (1976) (White group: *n* = 46)	0.03	0.20	6.7	1.1–39.9
Weissman (1976) (male group: *n* = 167)	0.11	0.23	2.1	1.2–3.6
Weissman (1976) (female group: *n* = 33)	0.002	0.10	50.0	0.02–110727.0
Weissman (1976) (13–17 onset group: *n* = 51)	0.00	0.23	–	–
Weissman (1976) (18–21 onset group: *n* = 83)	0.14	0.21	1.5	0.71–3.1
Weissman (1976) (22+ onset group: *n* = 66)	0.12	0.18	1.5	0.61–3.7

*Violence*				
Alexander 1974 (*n* = 101)	0.03	0.04	1.3	0.30–5.9
Anglin 1988 (White group: *n* = 275)	0.02	0.04	2.0	0.72–5.6
Anglin 1988 (Mexican American group: *n* = 228)	0.09	0.05	0.56	0.27–1.1
Cushman, 1974 (*n* = 210)	0.003	0.02	6.7	5.3–8.4
McGlothlin (1978) (1962–64 group: *n* = 277)	0.03	0.03	1.0	0.38–2.6
McGlothlin (1978) (1970 group: *n* = 175)	0.03	0.05	1.7	0.65–4.3
Mott 1974 (male group: *n* = 207)	0.04	0.04	1.03	0.41–2.6
Weissman (1974) (Black group: *n* = 113)^f^	0.11	0.08	0.68	0.29–1.6
Weissman (1974) (Hispanic group: *n* = 86)^f^	0.09	0.19	2.1	0.90–4.9
Weissman (1974) (White group: *n* = 77)^f^	0.05	0.33	6.1	2.2–17.3
Weissman (1974) (male group: *n* = 223)^f^	0.10	0.11	1.1	0.61–1.9
Weissman (1974) (female group: *n* = 53)^f^	0.03	0.50	16.0	3.3–77.4
Weissman (1974) (13–20 onset group: *n* = 109)^f^	0.10	0.19	2.0	0.94–4.1
Weissman (1974) (21–25 onset group: *n* = 99)^f^	0.06	0.27	4.6	1.9–11.4
Weissman (1974) (26+ onset group: *n* = 68)^f^	0.12	0.05	0.39	0.11–1.4
Weissman (1976) (Black group: *n* = 73)^f^	0.05	0.17	3.4	1.1–10.9
Weissman (1976) (Mexican American group: *n* = 81)^f^	0.06	0.08	1.3	0.47–3.8
Weissman (1976) (White group: *n* = 46)^f^	0.00	0.08	–	–
Weissman (1976) (male group: *n* = 167)^f^	0.05	0.14	2.8	1.3–6.2
Weissman (1976) (female group: *n* = 33)^f^	0.01	0.02	2.0	0.03–130.0
Weissman (1976) (13–17 onset group: *n* = 51)^f^	0.00	0.10	–	–
Weissman (1976) (18–21 onset group: *n* = 83)^f^	0.08	0.15	1.9	0.73–4.8
Weissman (1976) (22+ onset group: *n* = 66)^f^	0.04	0.10	2.5	0.60–10.4

*Robbery*				
Anglin 1988 (White group: *n* = 275)	0.02	0.03	1.5	0.51–4.4
Anglin 1988 (Mexican American group: *n* = 228)	0.04	0.03	0.88	0.28–2.0
Cushman, 1974 (*n* = 210)	0.01	0.03	4.6	3.7–5.9
McGlothlin (1978) (1962–64 group: *n* = 277)	0.04	0.02	0.50	0.18–1.4
McGlothlin (1978) (1970 group: *n* = 175)	0.02	0.10	5.0	1.6–15.8
Weissman (1974) (Black group: *n* = 113)	0.09	0.15	1.6	0.73–3.4
Weissman (1974) (Hispanic group: *n* = 86)	0.08	0.09	1.2	0.43–3.4
Weissman (1974) (White group: *n* = 77)	0.06	0.25	4.5	1.6–12.7
Weissman (1974) (male group: *n* = 223)	0.09	0.19	2.2	1.3–3.8
Weissman (1974) (female group: *n* = 53)	0.05	0.03	0.56	0.08–4.0
Weissman (1974) (13–20 onset group: *n* = 109)	0.10	0.09	0.93	0.39–2.2
Weissman (1974) (21–25 onset group: *n* = 99)	0.07	0.26	3.6	1.6–8.3
Weissman (1974) (26+ onset group: *n* = 68)	0.06	0.12	2.1	0.61–7.0
Weissman (1976) (Black group: *n* = 73)	0.06	0.08	1.3	0.39–4.6
Weissman (1976) (Mexican American group: *n* = 81)	0.02	0.08	4.0	0.72–22.4
Weissman (1976) (White group: *n* = 46)	0.05	0.10	2.0	0.41–9.8
Weissman (1976) (male group: *n* = 167)	0.04	0.09	2.2	0.90–5.6
Weissman (1976) (female group: *n* = 33)	0.07	0.04	0.57	0.07–4.8
Weissman (1976) (13–17 onset group: *n* = 51)	0.00	0.12	–	–
Weissman (1976) (18–21 onset group: *n* = 83)	0.05	0.07	1.4	0.40–4.9
Weissman (1976) (22+ onset group: *n* = 66)	0.06	0.07	1.2	0.30–4.5

*Prostitution*				
Cushman, 1974 (*n* = 210)	0.001	0.03	33.0	26.2–41.6
Weissman (1974) (male group: *n* = 223)	0.001	0.003	3.0	0.02–365.3
Weissman (1974) (female group: *n* = 53)	0.40	0.27	0.69	0.35–1.3
Weissman (1976)(Black group: *n* = 73)	0.001	0.11	110.0	0.08–160774.9
Weissman (1976) (Mexican American group: *n* = 81)	0.00	0.02	–	–
Weissman (1976) (White group: *n* = 46)	0.00	0.02	–	–
Weissman (1976) (male group: *n* = 167)	0.00	0.02	–	–
Weissman (1976) (female group: *n* = 33)	0.00	0.23	–	–
Weissman (1976) (13–17 onset group: *n* = 51)	0.00	0.02	–	–
Weissman (1976) (18–21 onset group: *n* = 83)	0.00	0.08	–	–
Weissman (1976) (22+ onset group: *n* = 66)	0.001	0.05	50.0	0.02–110727.0

a Calculated using estimated number of events if not reported.

b ‘money’ crime.

c Petty theft/forgery.

d Property felony.

e Crime against property.

f Assault.

g ‘breaking and entering’.

## References

[bib0005] Alexander M., McCaslin C. (1974). Criminality in heroin addicts before, during and after methadone treatment. American Journal of Public Health.

[bib0010] Altman D.G. (1991). Practical statistics for medical research.

[bib0015] Anglin M.D., Booth M.W., Ryan T.M., Hser Y.-I. (1988). Ethnic differences in narcotics addiction. II. Chicano and Anglo addiction career patterns. International Journal of Addiction.

[bib0020] Anglin M.D., Hser Y.-I. (1987). Addicted women and crime. Criminology.

[bib0025] Anglin M.D., Ryan T.M., Booth M.W., Hser Y.-I. (1988). Ethnic differences in narcotics addiction. I. Characteristics of Chicano and Anglo methadone maintenance clients. International Journal of Addiction.

[bib0030] Anglin M.D., Speckart G. (1988). Narcotics use and crime: A multisample, multimethod analysis. Criminology.

[bib0035] Asbridge M., Hayden J.A., Cartwright J.L. (2012). Acute cannabis consumption and motor vehicle collision risk: Systematic review of observational studies and meta-analysis. BMJ.

[bib0040] Beckett D., Lodge K.J. (1971). Aspects of social relationships in heroin addicts admitted for treatment. Bulletin on Narcotics.

[bib0045] Bennett T., Holloway K. (2005). The association between multiple drug misuse and crime. International Journal of Offender Therapy and Comparative Criminology.

[bib0050] Bennett T., Holloway K., Farrington D. (2008). The statistical association between drug misuse and crime: A meta-analysis. Aggression & Violent Behavior.

[bib0055] Blumstein A., Cohen J. (1987). Characterizing criminal careers. Science.

[bib0060] Bond J.W., Sheridan L. (2007). The relationship between the detection of acquisitive crime by forensic science and drug-dependent offenders. Journal of Forensic Science.

[bib0065] Borenstein M., Hedges L.V., Higgins J.P.T., Rothstein H.R. (2009). Introduction to meta-analysis.

[bib0070] Brownstein H.H., Brownstein H. H (2016). Drugs and violent crime. The handbook of drugs and society.

[bib0075] Bukten A., Skurtveit S., Gossop M., Waal H., Stangeland P., Havnes I. (2011). Engagement with opioid maintenance treatment and reductions in crime: A longitudinal national cohort study. Addiction.

[bib0080] Bukten A., Skurtveit S., Stangeland P., Gossop M., Willersrud A.B., Waal H. (2011). Criminal convictions among dependent heroin users during a 3-year period prior to opioid maintenance treatment: A longitudinal national cohort study. Journal of Substance Abuse Treatment.

[bib0085] Cicero T.J., Ellis M.S., Surratt H.L., Kurtz S.P. (2014). The changing face of heroin use in the United States. JAMA Psychiatry.

[bib0090] Chambers C.D., Hinesley R.K., Moldestad M. (1970). Narcotic addiction in females: A race comparison. International Journal of Addiction.

[bib0095] Comiskey C.M., Stapleton R., Kelly P.A. (2012). Ongoing cocaine and benzodiazepine use: Effects on acquisitive crime committal rates amongst opiate users in treatment. Drugs: Education, Prevention and Policy.

[bib0100] Coughlan S.S. (1990). Recall bias in epidemiologic studies. Journal of Clinical Epidemiology.

[bib0105] Cushman P. (1974). Relationship between narcotic addiction and crime. Federal Probation.

[bib0110] Darke S., Mills K.L., Ross J., Williamson A., Havard A., Teesson M. (2009). The ageing heroin user: Career length, clinical profile and outcomes across 36 months. Drug and Alcohol Review.

[bib0115] Deeks J.J., Dinnes J., D’Amico R., Sowden A.J., Sakarovitch C., Song F. (2003). Evaluating non-randomised intervention studies. Health Technology Assessment.

[bib0120] Deschenes E.P., Anglin M.D., Speckart G. (1991). Narcotics addiction: Related criminal careers, social and economic costs. Journal of Drug Issues.

[bib0125] Farabee D., Joshi V., Anglin M.D. (2001). Addiction careers and criminal specialization. Crime and Delinquency.

[bib0130] Galea S., Nandi A., Vlahov D. (2004). The social epidemiology of substance use. Epidemiologic Reviews.

[bib0135] Gauffin K., Vinnerljung B., Fridell M., Hesse M., Hjern A. (2013). Childhood socio-economic status, school failure and drug abuse: A Swedish national cohort study. Addiction.

[bib0140] Goldstein P. (1985). The drugs/violence nexus: A tripartite conceptual framework. Journal of Drug Issues.

[bib0145] Gordon A.M. (1973). Patterns of delinquency in drug addiction. British Journal of Psychiatry.

[bib0150] Greenland S., O’Rourke K., Rothman K.J., Greenland S., Lash T.L. (2008). Meta-analysis. Modern epidemiology.

[bib0155] Hall W., Bell J., Carless J. (1993). Crime and drug use among applicants for methadone maintenance. Drug and Alcohol Dependence.

[bib0160] Hammersley R., Forsyth A., Morrison V., Davies J.B. (1989). The relationship between crime and opioid use. Addiction.

[bib0165] Hayhurst K.P., Jones A., Millar T., Pierce M., Davies L., Weston S. (2013). Drug spend and acquisitive offending by substance misusers. Drug and Alcohol Dependence.

[bib0170] Hayhurst K.P., Leitner M., Davies L., Flentje R., Millar T., Jones A. (2015). The effectiveness and cost effectiveness of diversion and aftercare programmes for offenders using class A drugs: A systematic review and economic evaluation. Health Technology Assessment.

[bib0175] Government H.M. (2010). Drug Strategy 2010. Reducing demand, restricting supply, building recovery: Supporting people to live a drug free life.

[bib0180] Holloway K., Bennett T. (2007). Gender differences in drug misuse and related problem behaviors among arrestees in the UK. Substance Use and Misuse.

[bib0185] Home Office (2011). Drug interventions programme operational handbook. http://www.nta.nhs.uk/uploads/dip_operational_handbook.pdf.

[bib0190] Home Office (2016). Modernising crime prevention strategy. https://www.gov.uk/government/uploads/system/uploads/attachment_data/file/509831/6.1770_Modern_Crime_Prevention_Strategy_final_WEB_version.pdf.

[bib0195] Hser Y.-I., Anglin M.D., Booth M.W. (1987). Sex differences in addict careers. 3. Addiction. American Journal of Drug and Alcohol Abuse.

[bib0200] James I., D’Orban P.T. (1970). Patterns of delinquency among British heroin addicts. Bulletin on Narcotics.

[bib0205] James J., Gosho C., Wohl R.W. (1979). The relationship between female criminality and drug use. International Journal of Addiction.

[bib0210] Jarvis G., Parker H. (1989). Young heroin users and crime. How do the ‘new users’ finance their habits?. British Journal of Criminology.

[bib0215] Johnson H. (2006). Drug use by incarcerated women offenders. Drug and Alcohol Review.

[bib0220] Jones C.M. (2013). Heroin use and heroin use risk behaviors among nonmedical users of prescription pain relievers – United States, 2002–2004 and 2008–2010. Drug and Alcohol Dependence.

[bib0225] Kaye S., Darke S., Finlay-Jones R. (1998). The onset of heroin use and criminal behaviour: Does order make a difference?. Drug and Alcohol Dependence.

[bib0230] Kimber J., Larney S., Hickman M., Randall D., Degenhardt L. (2015). Mortality risk of opioid substitution therapy with methadone versus buprenorphine: A retrospective cohort study. Lancet Psychiatry.

[bib0235] Klee H., Morris J. (1994). Crime and drug misuse: Economic and psychological aspects of the criminal activities of heroin and amphetamine injectors. Addiction Research and Theory.

[bib0240] Larm P., Hodgins S., Larsson A., Samuelson Y.M., Tengstrom A. (2008). Long-term outcomes of adolescents treated for substance misuse. Drug and Alcohol Dependence.

[bib0245] Lillie-Blanton M., Anthony J.C., Schuster C.R. (1993). Probing the meaning of racial/ethnic group comparisons in crack cocaine smoking. Journal of the American Medical Association.

[bib0250] Lynskey M.T., Hall W. (1998). Cohort trends in age of initiation to heroin use. Drug and Alcohol Review.

[bib0255] Martin S.E., Maxwell C.D., White H.R., Zhang Y. (2004). Trends in alcohol use, cocaine use, and crime: 1989–1998. Journal of Drug Issues.

[bib0260] McGlothlin W.H., Anglin M.D., Wilson B.D. (1978). Narcotic addiction and crime. Criminology.

[bib0265] Moher D., Liberati A., Tetzlaff J., Altman D.G., Group PRISMA. (2009). Preferred reporting items for systematic reviews and meta-analyses: The PRISMA statement. Annals of Internal Medicine.

[bib0270] Mott J. (1975). The criminal histories of male non-medical opiate users in the United Kingdom. Bulletin on Narcotics.

[bib0275] Mott J., Rathod N.H. (1976). Heroin misuse and delinquency in a new town. British Journal of Psychiatry.

[bib0280] Mott J., Taylor M. (1974). Delinquency amongst opiate users.

[bib0285] Nurco D.N., DuPont R.L. (1977). A preliminary report on crime and addiction within a community-wide population of narcotic addicts. Drug and Alcohol Dependence.

[bib0290] Nurco D.N., Hanlon T.E., Balter M.B., Kinlock T.W., Slaght E. (1991). A classification of narcotic addicts based on type, amount and severity of crime. Journal of Drug Issues.

[bib0295] Nurco D.N., Shaffer J.W., Ball J.C., Kinlock T.W. (1984). Trends in the commission of crime among narcotic addicts over successive periods of addiction and nonaddiction. American Journal of Drug and Alcohol Abuse.

[bib0300] O’Donnell J.A. (1966). Narcotic addiction and crime. Social Problems.

[bib0305] Parker R.N., Auerhahn K. (1998). Alcohol, drugs, and violence. Annual Review of Sociology.

[bib0310] Parker H., Bakx K., Newcombe R. (1986). Drug misuse in Wirral: A study of eighteen hundred problem drug users known to official agencies.

[bib0315] Parker H., Newcombe R. (1987). Heroin use and acquisitive crime in an English community. British Journal of Sociology.

[bib0320] Parker H., Newcombe R., Bakx K. (1987). The new heroin users: Prevalence and characteristics in Wirral, Merseyside. Addiction.

[bib0325] Perry A.E., Darwin Z., Godfrey C., McDougall C., Lunn J., Glanville J. (2009). The effectiveness of interventions for drug-using offenders in the court, secure establishments and the community: A systematic review. Substance Use and Misuse.

[bib0330] Perry A.E., Neilson M., Martyn-St James M., Glanville J.M., Woodhouse R., Godfrey C., Hewitt C. (2015). Interventions for drug-using offenders with co-occurring mental illness. Cochrane Database of Systematic Reviews Issue.

[bib0335] PHE (2013). Adult drug statistics from the National Drug Treatment Monitoring System (NDTMS). 1 April 2012 to 31 March 2013.

[bib0340] PHE (2014). Adult drug statistics from the National Drug Treatment Monitoring System (NDTMS). 1 April 2013 to 31 March 2014. http://www.nta.nhs.uk/uploads/adult-drug-statistics-from-the-national-drug-treatment-monitoring-system-2013-14.pdf.

[bib0345] Pierce M., Bird S., Hickman M., Millar T. (2015). National record linkage study of mortality for a large cohort of opioid users ascertained by drug treatment or criminal justice sources in England, 2005–2009. Drug and Alcohol Dependence.

[bib0350] Pierce M., Hayhurst K., Bird S., Hickman M., Seddon T., Dunn G. (2015). Quantifying crime associated with drug use among a large cohort of sanctioned offenders in England and Wales. Drug and Alcohol Dependence.

[bib0355] Pudney S. (2002). The road to ruin? Sequences of initiation into drug use and offending by young people in Britain.

[bib0360] Research Triangle Institute (1976). Drug use and crime: Report of the panel on drug use and criminal behavior.

[bib0365] Seddon T. (2000). Explaining the drug-crime link: Theoretical, policy and research issues. Journal of Social Policy.

[bib0370] Shaffer J.W., Nurco D.N., Ball J.C., Kinlock T.W. (1985). The frequency of nonnarcotic drug use and its relationship to criminal activity among narcotic addicts. Comprehensive Psychiatry.

[bib0375] Shaffer J.W., Nurco D.N., Ball J.C., Kinlock T.W., Duszynski K.R., Langrod J. (1987). The relationship of preaddiction characteristics to the types and amounts of crime committed by narcotic addicts. International Journal of Addiction.

[bib0380] Shand F.L., Slade T., Degenhardt L., Baillie A., Nelson E.C. (2011). Opioid dependence latent structure: Two classes with differing severity?. Addiction.

[bib0385] Stenbacka M., Stattin H. (2007). Adolescent use of illicit drugs and adult offending: A Swedish longitudinal study. Drug and Alcohol Review.

[bib0390] Strug D., Wish E., Johnson B., Anderson K., Miller T., Sears A. (1984). The role of alcohol in the crimes of active heroin users. Crime and Delinquency.

[bib0395] Swan A.C., Goodman-Delahunty J. (2013). The relationship between drug use and crime among police detainees: Does gender matter?. International Journal of Forensic Mental Health.

[bib0400] Sweeten G., Piquero A.R., Steinberg L. (2013). Age and the explanation of crime, revisited. Journal of Youth and Adolescence.

[bib0405] Toma M., McAlister F.A., Bialy L., Adams D., Vandermeer B., Armstrong P.W. (2006). Transition from meeting abstract to full-length journal article for randomized controlled trials. JAMA.

[bib0410] UNODC (2015). World drug report 2015.

[bib0415] Vecchi S., Belleudi V., Amato L., Davoli M., Perucci C.A. (2009). Does direction of results of abstracts to scientific conferences on drug addiction predict full publication?. BMC Medical Research Methodology.

[bib0420] Vorenberg J., Lukoff I.F. (1973). Addiction, crime and the criminal justice system. Federal Probation.

[bib0425] Weissman J.C., Katsampes P.L., Giacinti T.A. (1974). Opiate use and criminality among a jail population. Addictive Diseases.

[bib0430] Weissman J.C., Marr S.W., Katsampes P.L. (1976). Addiction and criminal behavior: A continuing examination of criminal addicts. Journal of Drug Issues.

[bib0435] Wells G., Shay B. (2000). Data extraction for nonrandomised systematic reviews.

[bib0440] Wiepert G.D., D’Orbán P.T., Bewley T.H. (1979). Delinquency by opiate addicts treated at two London clinics. British Journal of Psychiatry.

[bib0445] Wright S., Klee H. (2001). Violent crime, aggression and amphetamine: What are the implications for drug treatment services?. Drugs: Education, Prevention and Policy.

