# Supplementary material for: Pathways through opiate use and offending: A systematic review
Source: Int J Drug Policy. 2017 Jan;39:1–13. doi: 10.1016/j.drugpo.2016.08.015 (PMC5234472; doi:10.1016/j.drugpo.2016.08.015)
Supplement: Supplementary file 1 [file mmc1.docx]

**Supplementary Table 1** Search strategy.

| adolescents/ OR age of onset/ OR cohort analysis/ OR critical pathways/ OR event history analysis/ OR followup studies/ OR longitudinal studies/ OR personal history/ OR prospective studies/ OR time course/ OR time to event data/ OR young adult offenders/ OR young people/ OR (age NEAR/4 crim*) OR (criminal NEAR/4 career) OR (event NEAR/2 history) OR (life NEAR/2 (course OR cycle OR span OR time)) OR (natural NEAR/2 histor*) OR (survival NEAR/2 (analys* OR method*)) OR (young NEAR/2 (adult* OR people OR person)) OR (time NEAR/2 to NEAR/2 event) OR desistence OR duration OR juvenile* OR pathway* OR persistence OR route* OR sequence* OR temporal OR timing* OR trajector* OR typolog*  **AND**  cocaine/ OR crack/ OR drug abuse/ OR drug addiction/ OR drug dependency/ OR heroin/ OR methadone/ OR narcotics/ OR opiates/ OR opioids/ OR substance abuse/ OR substance abusers/ OR substance abuse disorders/ OR substance dependency/ OR (multiple NEAR/2 drug*) OR (poly NEAR/2 drug*) OR (poly NEAR/2 substance*) OR (problem* NEAR/2 drug*) OR (cocaine OR crack OR drug* OR heroin OR methadone OR narcotic* OR opiate* OR opioid* OR substance*) NEAR/2 (abuse* OR addict* OR dependen* OR misuse* OR use OR user)  **AND**  arrests/ OR burglary/ OR crime/ OR crime control/ OR crime prevention/ OR crime rate/ OR criminal behaviour/ OR criminal careers/ OR criminal damage/ OR criminal justice/ OR criminal justice system/ OR criminal law/ OR criminal offences/ OR criminology/ OR dangerous driving/ OR delinquency/ OR deviant behaviour/ OR drug dealing/ OR drug offences/ OR forgery/ OR fraud/ OR handling stolen goods/ OR juvenile crime/ OR juvenile justice/ OR juvenile justice system/ OR juvenile offenders/ OR legal system/ OR offences against the person/ OR offenders/ OR prisoners/ OR prison service/ OR probationers/ OR recidivists/ OR remand offenders/ OR remand prisoners/ OR robbery/ OR sexual offences/ OR shoplifting/ OR street crime/ OR street violence/ OR theft/ OR violent crime/ OR violent juvenile offenders/ OR violent offenders/ OR violent street crime/ OR young adult offenders/ OR young offenders/ OR ((criminal OR juvenile OR youth) NEAR/2 (justice)) OR (arrest* NEAR/2 refer*) OR (drug* NEAR/2 test*) OR (secure NEAR/2 (establishment* OR facilit*) OR (trigger NEAR/2 offen*) OR (community NEAR/2 sentence*) OR (drug* NEAR/2 intervention* NEAR/2 program*) OR acquisitive OR arrest* OR convict* OR correction* OR court* OR crime OR criminal* OR custod* OR delinquen* OR detain* OR detention* OR deviance OR deviancy OR deviant* OR divert* OR diversion* OR DTTO* OR felon* OR imprison* OR incarcerat* OR inmate* OR jail* OR offence* OR offender* OR parole* OR penal OR penitentia* OR prison* OR probation* OR punish* OR recidivis* OR referral OR reincarcerat* OR remand* OR reoffend* |
| --- |
