# Supplementary figures and images for: Pathways through opiate use and offending: A systematic review
Source: Int J Drug Policy. 2017 Jan;39:1–13. doi: 10.1016/j.drugpo.2016.08.015 (PMC5234472; doi:10.1016/j.drugpo.2016.08.015)

**Supplementary Fig. 1.** Rate ratios: theft*.
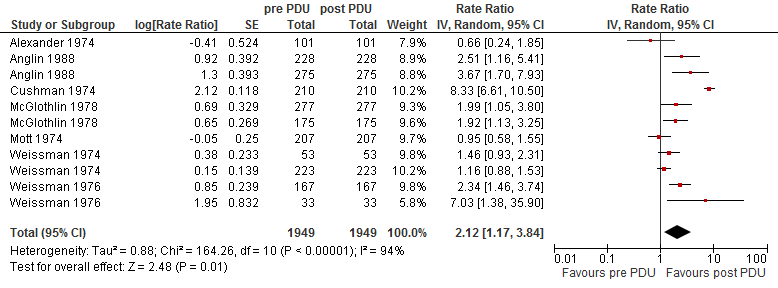


* Note: all paired studies

Supplement: Supplementary file 2 [file mmc2.docx]

**Supplementary Fig. 2.** Rate ratios: burglary.*

**
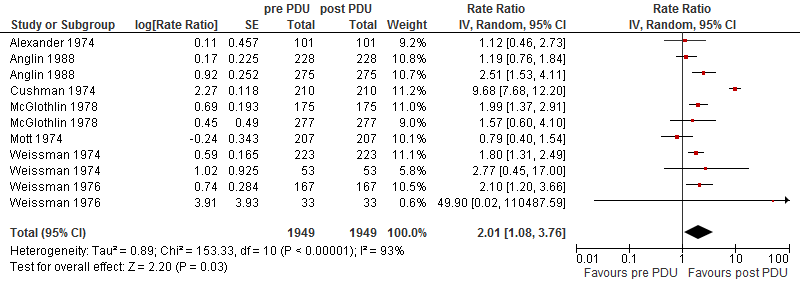
**

* Note: all paired studies

Supplement: Supplementary file 3 [file mmc3.docx]
